# Differential Diagnosis of Infectious Versus Autoimmune Encephalitis Using Artificial Intelligence-Based Modeling

**DOI:** 10.3390/jcm14228222

**Published:** 2025-11-20

**Authors:** David Petrosian, Nataša Giedraitienė, Vera Taluntienė, Dagnė Apynytė, Haroldas Bikelis, Gytis Makarevičius, Mantas Jokubaitis, Mantas Vaišvilas

**Affiliations:** 1 Faculty of Medicine, Vilnius University, Vilnius 03101, Lithuania; 2 Faculty of Medicine, Institute of Clinical Medicine, Vilnius University, Vilnius 03101, Lithuania

**Keywords:** autoimmune encephalitis, infectious encephalitis, paraneoplastic neurologic syndromes, artificial intelligence, machine learning

## Abstract

**Background**: Encephalitis is a severe and potentially life-threatening inflammatory disorder of the central nervous system. Without prompt diagnosis and appropriate treatment, it often results in poor clinical outcomes. The study aimed to develop an artificial intelligence-based model that distinguishes autoimmune encephalitis from infectious encephalitis, encompassing a broad spectrum of autoimmune encephalitis phenotypes, serostatuses, and neuroimmunological entities. **Methods**: We conducted a retrospective analysis of patients diagnosed with autoimmune encephalitis, including paraneoplastic neurological syndromes and/or infectious encephalitis, at Vilnius University Hospital Santaros Klinikos from 2016 to 2024. Supervised machine learning techniques were used to train the models, and Shapley Additive Explanations analysis was applied to improve their interpretability. **Results**: A total of 233 patients were included in the study. The Random Forest model demonstrated the best performance in differentiating the etiology of encephalitis, achieving an AUROC of 0.966. Further analysis revealed that laboratory, electroencephalography, and clinical data were the most influential predictors, whereas imaging data contributed less to classification accuracy. **Conclusions**: We developed a machine learning model capable of distinguishing infectious encephalitis from both seropositive and seronegative autoimmune encephalitis. Since autoimmune cases may be misdiagnosed as infectious in the absence of detectable antibodies, our model has the potential to support clinical decision-making and reduce diagnostic uncertainty.

## 1. Introduction

Encephalitis is a life-threatening inflammatory nervous system condition with poor overall outcomes without timely diagnosis and appropriate management [1,2]. The two most common forms of encephalitis are infectious (IE) and antibody-positive autoimmune encephalitis (AE), with a comparable incidence of ~1/100,000 person/years [3].

Although encephalitis is rare, prompt etiological diagnosis is essential, as delayed treatment delays frequently result in persistent neurological sequelae [4]. Unfortunately, establishment of etiological diagnosis remains challenging. Even in tertiary academic centers equipped with advanced diagnostic capabilities, including brain biopsies, the etiology of encephalitis remains undetetermined in 30% to 60% of cases [5,6,7]. Furthermore, in routine clinical settings, the diagnostic yield is likely even lower due to the limited availability of advanced investigative tools. Commercially available polymerase chain reaction assays (PCR) for IE detect only a limited number of pathogens and may produce false-negative results due to various factors [8,9,10,11]. Similarly, the availability of commercial autoantibody testing for AE is limited, costly, and frequently associated with diagnostic inaccuracies regardless of diagnostic modality [12,13,14,15].

To address these limitations, multiple scoring systems have been proposed to either diagnose AE or differentiate between AE and IE, many of which have demonstrated high performance and external validation [16,17]. In contrast, artificial intelligence (AI)-based techniques have rarely been explored. A few published studies demonstrated initial results suggesting that AI may differentiate the etiology of encephalitis with accuracy comparable to that of experienced neurologists [18,19]. However, these studies have involved a relatively small sample size of autoimmune encephalitis (AE), with a primary focus on antibody-positive limbic encephalitis. In addition, they have not addressed extra-limbic central nervous system manifestations, seronegative AE, peripheral nervous system involvement, or paraneoplastic neurological syndromes. Moreover, most previous models have relied primarily on MRI or laboratory data.

To build on this research, we aimed to develop a model that distinguishes between AE and IE, incorporating a wider spectrum of AE phenotypes, serostatuses, and neuroimmunological entities. Importantly, our approach integrates both clinical and laboratory data to enhance its applicability in real-world clinical practice.

## 2. Materials and Methods

### 2.1. Data Collection

We retrospectively collected data on patients diagnosed with AE including paraneoplastic neurological syndromes (PNS) and/or IE from Vilnius University Hospital Santaros Klinikos between 2016 and 2024. For AE and PNS and their respective clinical syndromes, diagnosis was made based on published relevant guidelines [20,21,22,23]. For other immune-mediated neuroinflammatory conditions, diagnosis was made using established criteria with histological verification when available [24]. Antibody testing was performed with commercially available indirect immunofluorescence cell-based assays (CBA; Euroimmun, Lubeck, Germany) for the detection of neuronal surface antibodies (anti-N-methyl-D-aspartate receptor (NMDAR), anti-leucine-rich glioma-inactivated protein 1 (LGI-1), anti-α-amino-3-hydroxy-5-methyl-4-isoxazolepropionic acid receptor (AMPAR), anti-contactin-associated protein 2 (CASPR2), anti-gamma-amino-butyric acid B-receptor (GABAbR)) were used in accordance with the manufacturer’s instructions. For commercial CBAs, both serum and cerebrospinal fluid (CSF) were tested when available. Lineblots for intracellular antibodies against intracellular antigens (anti-Hu, Anti-Yo, anti-Ma1/2, anti-amphiphisin, anti-DNER, anti-CV2, anti-titin, anti-recoverin, anti-GAD65; Euroimmun, Lubeck, Germany) were used in accordance with the manufacturer’s instructions. Paired CSF and serum testing was performed when feasible. Additionally, samples were screened using in-house rat brain immunohistochemistry as previously described [25] to detect autoantibodies beyond the scope for commercial assays.

For IE, cases with a confirmed pathogen were diagnosed based on either bacterial cultures (blood and/or CSF), CSF polymerase chain reaction (PCR), or both. In a minority of cases, pathogen-specific antibodies were identified using ELISA or Western blotting, with the demonstration of intrathecal antibody production in all cases when appropriate. A small fraction of patients received the diagnosis of IE only after brain biopsy.

The dataset included patient demographics, presenting symptoms, serum and CSF parameters, electroencephalographic (EEG) findings (including diffuse slowing/non-epileptic or epileptiform abnormalities), and MRI results (with or without contrast use). Presenting symptoms were defined as the patient’s major complaints at the time of admission and/or objective neurological findings after neurological or psychiatric evaluation. To increase statistical power, we incorporated an external dataset comprising 20 cases of LGI1-antibody encephalitis and 21 cases of herpes simplex virus encephalitis from Müller-Jensen et al. [26]. Prior to integration, we performed data harmonization to ensure consistency between the internal and external datasets. All variables were reviewed for alignment in definitions, measurement units, and coding practices. Categorical variables—such as clinical symptoms, EEG findings, and MRI abnormalities—were compared across datasets and assigned to shared categories based on equivalent clinical meaning. Continuous variables, including serum C-reactive protein (CRP), white blood cell (WBC) count, and CSF parameters, were checked for unit consistency and converted when required. Only variables that could be consistently aligned across both datasets were included in the combined analysis. The external dataset contained complete information for all required variables, including age, sex, clinical symptoms, serum CRP and WBC counts, CSF profiles, EEG results, and MRI findings.

### 2.2. Data Pre-Processing

Categorical variables were transformed into binary format using one-hot encoding. Missing values were handled via univariate imputation: continuous variables were imputed with their median values, while categorical variables were imputed using the mode of each class. The dataset was split into training (70%) and testing (30%) sets using stratified sampling to preserve the proportion of AE and IE cases. To address class imbalance, higher weights were assigned to the minority class during model training, thereby improving the sensitivity and overall performance.

Prior to model development, Recursive Feature Elimination with Cross-Validation (RFECV) was applied to identify the most informative predictors. RFECV was implemented using a stratified 5-fold cross-validation strategy. Feature elimination was guided by the AUROC metric, and the process continued until performance no longer improved or a minimum of 25 features remained. An XGBoost classifier was used as the underlying estimator due to its ability to handle nonlinear relationships and mixed data types.

### 2.3. Model Development and Validation

Supervised machine learning classifiers were employed to develop predictive models capable of distinguishing AE from IE. The models included XGBoost, Random Forest, LightGBM, Logistic Regression, K-Nearest Neighbors, and Gaussian Naïve Bayes.

Hyperparameter tuning for all models was performed using RandomizedSearchCV with stratified 5-fold cross-validation, which preserves the proportion of AE and IE cases in each fold. The AUROC metric was used to evaluate model performance during cross-validation, and the best hyperparameters were selected based on this score (see Table S1 for the full hyperparameter search spaces).

Models were evaluated on the independent test set that was retained during the dataset-splitting process. Performance was assessed using accuracy, sensitivity, specificity, F1-score, precision, and AUROC. SHAP (SHapley Additive exPlanations) was used to interpret the contribution of each variable to model predictions and identify the most important features driving differential diagnosis.

### 2.4. Statistical Analysis

Statistical analysis was conducted using R 4.4.3 (R Foundation for Statistical Computing, Vienna, Austria) and Python 3.11.4 (Python Software Foundation, Wilmington, DE, USA). The Shapiro–Wilk test was applied to check for data normality. Continuous data were presented as medians with interquartile ranges, and categorical data as frequencies along with percentages. Association between categorical variables was assessed using the chi-square test or Fisher’s exact test. Comparisons of two independent groups were conducted using the Mann–Whitney U test or independent samples *t*-test. Data visualization comprised pie charts to demonstrate the distribution of categorical variables, and chord diagrams created with the R ‘circlize’ package to illustrate relationships between variables. Locally weighted scatterplot smoothing (LOWESS) and zero-crossing analysis were applied to determine model-derived thresholds for laboratory features. Statistical significance was set at *p* < 0.05.

## 3. Results

### 3.1. Clinical and Paraclinical Features of the Cohort

Out of 368 total cases, we excluded 18 patients with hematologic malignancies (12 leukemia, 5 lymphoma, 1 myeloma), 10 with brain abscess, 6 with other oncologic conditions, and 101 with miscellaneous diagnoses not related to encephalitis, leaving a final cohort of 233 patients. The study included 233 patients (83/233 (35.6%) AE and 150/233 (64.4%) IE). Most cases in the autoimmune group consisted of antibody positive limbic encephalitis, while the infectious group included both viral (*n* = 84, 56.0%) and bacterial (*n* = 66, 44.0%) agents (Table 1).

Clinical, paraclinical, and demographical data are displayed in Table 2. Most common presenting symptoms for IE were fever (*n* = 110, 73.3%), ataxia (*n* = 62, 41.3%), and headache (*n* = 69, 46.0%). In contrast, seizures (*n* = 42, 50.6%), memory impairment (*n* = 37, 44.6%), emotional changes (*n* = 27, 32.5%), and behavioral changes (*n* = 28, 33.7%) were more frequent in AE. Laboratory findings showed that patients with IE had significantly higher CSF cell counts, protein levels, hypoglycorrhachia, and elevated serum CRP levels compared to AE.

Paraclinical findings were significant for encephalopathic EEG pattern in IE compared to AE and for parenchymal/meningeal contrast enhancement, mass effect, and restricted diffusion on MRI (Table 2).

**Figure 1:**
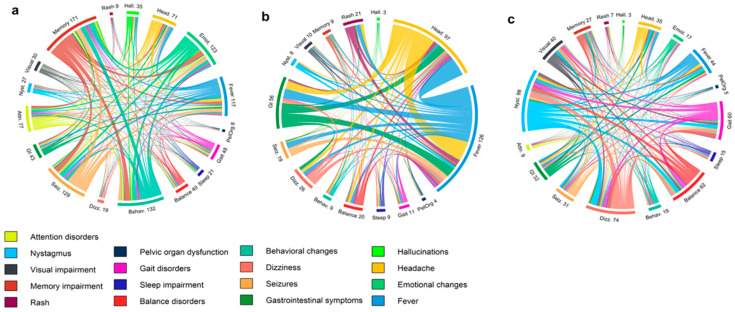
Chord diagrams illustrating symptom interconnections in (**a**) limbic encephalopathy, (**b**) generalized encephalopathy, and (**c**) cerebellar-brainstem syndrome.

**Figure 2:**
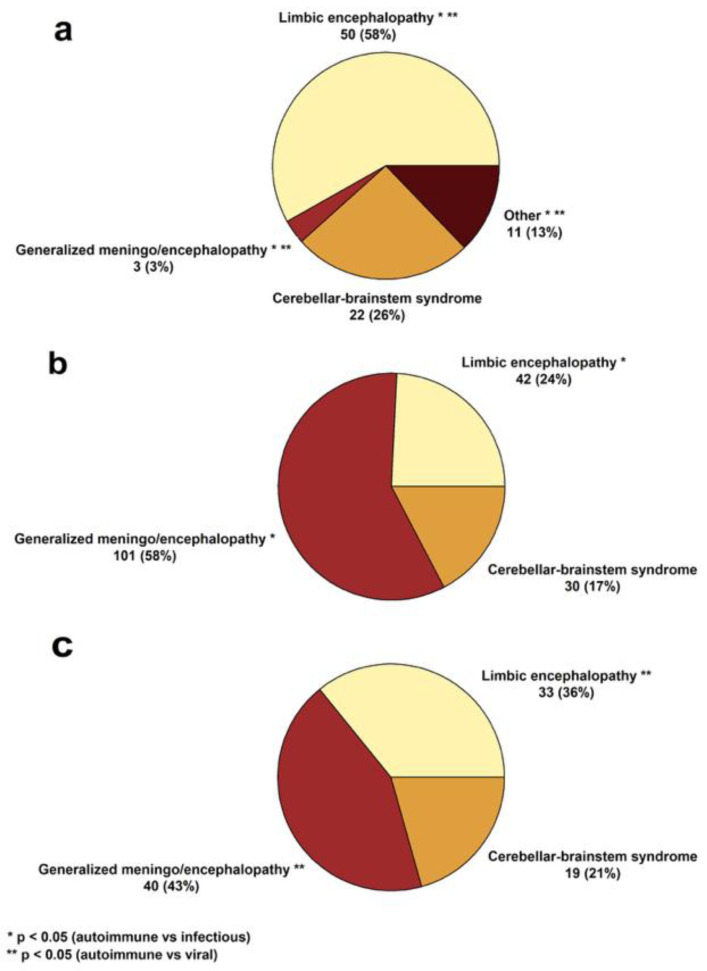
Pie charts depicting established clinical syndromes in (**a**) autoimmune, (**b**) infectious, and (**c**) viral encephalitis. The ‘Other’ category includes cases of encephalomyelitis, cerebral cortical encephalitis, CLIPPERS, diencephalitis, and encephalomyeloradiculoneuritis.

The most frequently observed clinical syndrome in AE was focal limbic encephalopathy, whereas generalized encephalopathy predominated in IE. Presenting symptoms, their relations, and established syndromes are illustrated in Figure 1 and Figure 2.

### 3.2. AI Modeling

After performing recursive feature elimination to select the most important variables, we identified a subset of features for model development. The features selected for AI modeling are presented in Table 3.

**Figure 3:**
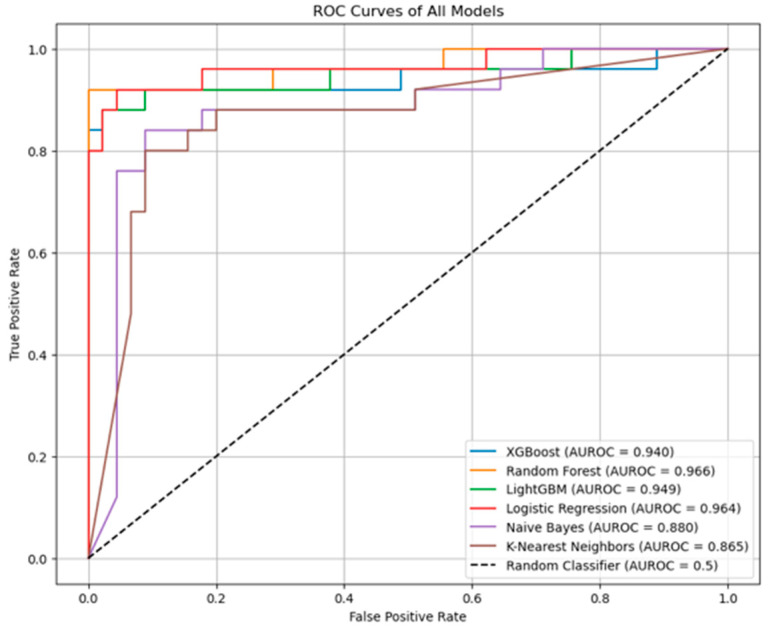
Receiver Operating Characteristic (ROC) curves comparing the performance of multiple machine learning models.

Using these variables, we employed a set of widely used classifiers—XGBoost, Random Forest, LightGBM, Logistic Regression, Naïve Bayes, and K-nearest Neighbors—chosen for their established performance in classification tasks. Random Forest was chosen as the final model because it was the most robust classifier with the highest predictive performance based on AUROC values. The DeLong test was performed to compare the AUROC values of the developed models, revealing that Random Forest, Logistic Regression, and LightGBM were more robust compared to K-Nearest Neighbors. No other statistically significant differences were found. Detailed performance metrics are presented in Table 4. AUROC values for different classifiers are shown in Figure 3.

**Figure 4:**
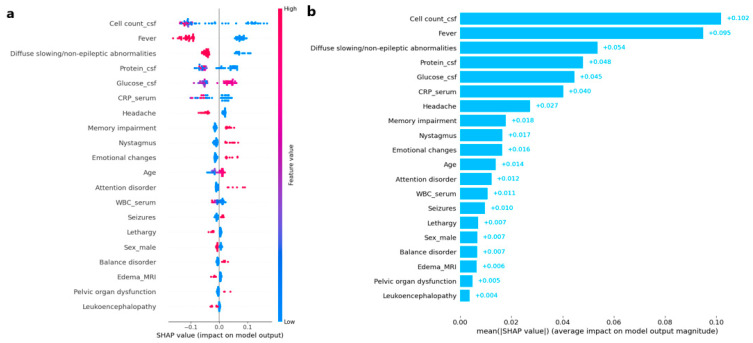
(**a**) SHAP beeswarm plot displaying the distribution and impact of individual feature values on the model output. Each dot represents a single instance’s SHAP value for a feature, colored by the feature value. (**b**) SHAP feature importance plot.

The SHAP framework was employed to evaluate the impact of different variables on the differential diagnosis in the developed machine learning model. Persistent fever (defined as body temperature ≥ 38 °C lasting ≥ 3 consecutive days), headache, and diffuse slowing/non-epileptic abnormalities on EEG were strongly associated with IE (Figure 4), whereas memory impairment, nystagmus, emotional changes, and seizures were predictive of AE. Among the laboratory features, elevated CSF cell counts and protein levels exhibited the highest predictive value for IE. In comparison, imaging features consistently demonstrated lower predictive importance, as quantified by their SHAP values relative to laboratory and clinical variables.

**Figure 5:**
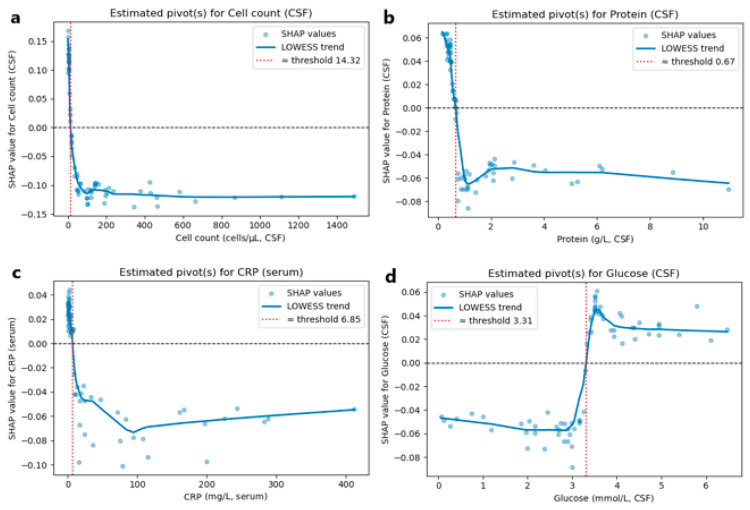
SHAP value–feature plots with LOWESS smoothing for CSF and serum biomarkers. Each panel displays SHAP values from the predictive model plotted against their respective feature values: (**a**) CSF cell count, (**b**) CSF protein, (**c**) serum CRP, and (**d**) CSF glucose. The blue line shows the LOWESS-smoothed trend. The vertical red line indicates the threshold where feature values begin to drive predictions toward autoimmune encephalitis diagnosis.

LOWESS smoothing of SHAP values was applied to identify zero-crossing points, which serve as model-derived thresholds beyond which the likelihood of autoimmune encephalitis begins to increase for key laboratory features: CSF cell count 14.32 cells/µL, CSF protein 0.67 g/L, serum CRP 6.85 mg/L, and CSF glucose 3.31 mmol/L (Figure 5).

Additionally, Figure S1 presents four force plots, each corresponding to a patient, including two seropositive and two seronegative cases, to demonstrate how individual features contributed to the diagnosis of AE in each case.

### 3.3. Comparison with Human Controls

**Figure 6:**
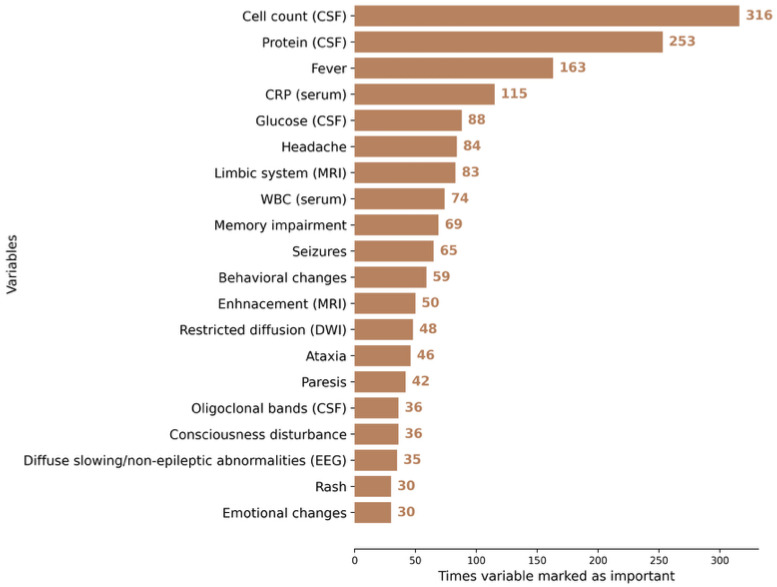
Variables most frequently identified as important by neurologists across all cases. CSF: cerebrospinal fluid, MRI: magnetic resonance imaging, DWI: diffusion-weighted imaging, EEG: electroencephalopgraphy.

To compare the performance of the developed AI model against human controls, we constructed an independent database comprised of 70 IE and AE cases that had not been used in model training. Model performance was then compared with that of clinicians (Table 5). The features made available to clinicians are detailed in Table 3. Further analysis demonstrated that clinicians primarily relied on laboratory data when differentiating encephalitis etiology. The features considered most impactful for the decision-making process are illustrated in Figure 6.

## 4. Discussion

In this study, we developed and evaluated an AI-based model to differentiate AE from IE using demographic, clinical, and paraclinical variables. The model incorporated both antibody-positive and antibody-negative cases as well as a substantial proportion of extra-limbic manifestations including peripheral nervous system disorders, thereby reflecting real-life clinical scenarios. Our model demonstrated good performance metrics with accuracy comparable to trained neurologists.

Previous studies have emphasized the time of symptom onset as a key discriminator between IE and AE [27,28]. Indeed, common subtypes of AE (e.g., LGI-1, CASPR2, GAD65 mediated syndromes) typically follow an indolent course [29,30,31]. This complicates diagnosis as current clinical criteria only account for cases with sub-acute symptom onset consequently delaying recognition and immunotherapy initiation. Our findings suggest that differential diagnosis may be achievable based on initial presenting symptoms, independent of disease course, with the potential for the earlier prospective identification of AE.

Serological testing remains the cornerstone of AE diagnosis, and the incorporation of seropositive cases into our model facilitated robust disease modeling despite known caveats of commercial assays. However, seronegative AE continues to pose substantial diagnostic challenges, often leading to misdiagnosis, delays in treatment, and worse clinical outcomes [32,33,34]. Although advanced techniques like phage immunoprecipitation sequencing may serve as an additional diagnostic modality in selected AE cases, they remain inaccessible to most clinical centers [35]. Since up to 50% of AE cases may be seronegative, there is a critical need for diagnostic tools that do not rely on antibody detection. Our results indicate that an AI-based approach may help to address this gap [19,36]. Future collaborative studies should prioritize the inclusion of seronegative cohorts as such an approach would be cost-effective and broadly applicable to real-world clinical settings.

In the testing of our model against neurologists and neurologists in training, we found that clinicians relied on laboratory features, particularly CSF cell count and protein levels. Despite selecting similar features to base the diagnosis, none of the human controls outperformed the model. Nevertheless, the AI-based model and human diagnosticians misclassified different cases. This suggests that etiological decisions of the AI and human controls are based on different data. For example, CSF cell count thresholds differentiating AE and IE differed greatly in our study from previous reports [27], likely due to differences in population characteristics and sample size, underscoring the limited generalizability of rigid cut-offs. In most cases, clinicians misclassified patients who did not present with typical features of a specific encephalitis etiology. Borderline laboratory values often contributed to these diagnostic errors. For example, some patients exhibited CSF pleocytosis, elevated cell counts, and increased protein levels, which could be misinterpreted as indicative of an infectious etiology. Differentiation remains challenging when laboratory findings do not clearly align with the presenting symptoms. In contrast, the SHAP framework provided insights into model decision-making, suggesting that AI can move beyond subjective thresholds such as cell count or symptom onset to refine etiological classification.

Likewise, MRI interpretation using AI-based techniques has shown potential to aid in the diagnosis of AE [37]. Radiomics techniques can extract quantitative features—such as texture, shape, and intensity—from medical images that may not be easily detected by the human eye [38]. While incorporating radiomics features could potentially improve model performance, we did not include them in the current study due to the relatively modest sample size and the high dimensionality of radiomics data. Including hundreds of imaging features with a limited number of AE cases could increase the risk of overfitting and reduce model generalizability. Future studies with larger, multi-center cohorts and external validation are warranted to investigate the added value of radiomics in enhancing the AI-based differentiation of AE and IE.

The major strength of this study is the integration of both seropositive and seronegative AE cases. Such an approach enhanced the model’s relevance for real-world practice, where autoimmune etiologies may be overlooked in the absence of antibody detection. Furthermore, the infectious etiology subgroup consisted of both bacterial and viral agents broadening the applicability and generalizability of the developed model. A deliberate methodological choice was made to exclude time of symptom onset, a parameter often used in clinical reasoning but limited by the indolent course of common AE subtypes (e.g., LGI1, CASPR2). By focusing on initial presenting features, our model is better positioned to facilitate early diagnosis.

The study also has limitations. First, our dataset sample size was modest, which may limit the robustness of the model, particularly for rare AE subtypes. A small sample size can reduce the generalizability of model-derived thresholds and increase the risk of overfitting to patterns present in the internal cohort. Second, we faced class imbalance, which was addressed using class weighting; however, this approach may accentuate overfitting to more prevalent classes, potentially affecting predictions in underrepresented patient groups. Third, external validation was not feasible in this study, and future investigations across independent populations are required to confirm the model’s performance and generalizability. Fewer seronegative cases were included than seropositive cases in the current dataset, which may limit predictive performance for this subgroup; nevertheless, their inclusion enhances the overall applicability of the model, and future studies with larger seronegative cohorts could further improve the predictions. Finally, the internal dataset originated from a single tertiary center (Vilnius University Hospital Santaros Klinikos), which may differ from other clinical settings in terms of patient demographics, comorbidities, and diagnostic practices. Consequently, the model’s applicability to community hospitals or resource-limited settings—where EEG or MRI may not be available—may be limited.

## 5. Conclusions

AI-based techniques can effectively distinguish between autoimmune encephalitis and infectious encephalitis in a manner comparable to human assessments based on presenting symptoms, without relying on the timing of symptom onset. The developed model encompasses both limbic and extralimbic cases, addressing scenarios of both antibody-positive and antibody-negative patients to better reflect real-world clinical situations. To account for the diagnostic gap of seronegative autoimmune encephalitis, future efforts in AI-based modeling should prioritize seronegative cases to address this critical clinical need and enhance diagnostic accuracy across the entire spectrum of autoimmune encephalitis.

## Figures and Tables

**Table 1:** Distribution of autoimmune and infectious encephalitides cases.

Autoimmune Encephalitides (*n* = 83)		Infectious Encephalitides (*n* = 150)	
Associated Antibody	*n* (%)	Associated Agent	*n* (%)
Anti-LGI1	29 (34.9%)	Viral	84 (56.0%)
Anti-NMDA	9 (10.8%)	HSV-1/HSV-2	34 (22.7%)
Anti-AQP4	9 (10.8%)	Unidentified	25 (16.7%)
Seronegative	9 (10.8%)	VZV	12 (8.0%)
Anti-Yo	7 (8.4%)	TBEV	10 (6.7%)
Anti-GAD65	4 (4.8%)	CMV	1 (0.7%)
Anti-CASPR2	3 (3.6%)	EBV	1 (0.7%)
Anti-Hu ^†^	3 (3.6%)	Parvovirus B19	1 (0.7%)
Anti-AMPAR	2 (2.4%)	Bacterial	66 (44.0%)
Atypical	2 (2.4%)	Unidentified	31 (20.7%)
Anti-GABAB	1 (1.2%)	*Streptococcus* spp.	8 (5.3%)
Anti-KLHL11	1 (1.2%)	*L. monocytogenes*	8 (5.3%)
Anti-GFAP	1 (1.2%)	*B. burgdorferi*	7 (4.7%)
Anti-Ri	1 (1.2%)	*Staphylococcus* spp.	5 (3.3%)
Anti-MOG	1 (1.2%)	*N. meningitidis*	3 (2.0%)
ANA	1 (1.2%)	*M. tuberculosis*	2 (1.3%)
		*H. influenzae*	1 (0.7%)
		*T. pallidum*	1 (0.7%)

NMDA: N-methyl-D-aspartate receptor; LGI1: leucine-rich glioma inactivated 1; CASPR2: contactin-associated protein-like 2; AMPAR: α-amino-3-hydroxy-5-methyl-4-isoxazolepropionic acid receptor; AQP4: aquaporin-4; MOG: myelin oligodendrocyte glycoprotein; GAD65: glutamic acid decarboxylase 65; KLHL11: Kelch-like protein 11; GFAP: glial fibrillary acidic protein; GABAB: gamma-aminobutyric acid type B receptor; ANA: anti-nuclear antibodies; VZV: varicella zoster virus; HSV: herpes simplex virus; TBEV: tick-borne encephalitis virus; CMV: cytomegalovirus; EBV: Epstein–Barr virus. ^†^ One anti-Hu case overlapped with anti-CV2 antibodies.

**Table 2:** Comparison of clinical features, laboratory data, EEG findings, and MRI abnormalities among patients with autoimmune, infectious, and viral encephalitis.

Variable	Autoimmune (*n* = 83)	Infectious ^†^ (*n* = 150)	Viral (*n* = 84)	*p*-Value *	*p*-Value **
Age (years), median (IQR)	59 (41–68.5)	46.5 (28–63)	54 (34.25–66)	0.0108	0.2032
Sex (male), *n* (%)	38 (45.8%)	88 (58.7%)	49 (58.3%)	0.0588	0.1045
Presenting symptoms					
Headache	5 (6.0%)	69 (46.0%)	42 (50.0%)	<0.0001	<0.0001
Disorientation	26 (31.3%)	45 (30.0%)	28 (33.3%)	0.8333	0.7815
Gait disturbance	17 (20.5%)	11 (7.3%)	7 (8.3%)	0.0031	0.0252
Sleep impairment	7 (8.4%)	3 (2.0%)	2 (2.4%)	0.0370	0.0989
Behavioral changes	28 (33.7%)	13 (8.7%)	12 (14.3%)	<0.0001	0.0032
Balance disorder	17 (20.5%)	10 (6.7%)	7 (8.3%)	0.0016	0.0252
Fever	10 (12.0%)	110 (73.3%)	62 (73.8%)	<0.0001	<0.0001
Impaired consciousness	22 (26.5%)	35 (23.3%)	23 (27.4%)	0.5895	0.8986
Seizures	42 (50.6%)	31 (20.7%)	20 (23.8%)	<0.0001	0.0003
Paresthesia	9 (10.8%)	12 (8.0%)	6 (7.1%)	0.4680	0.4030
GI symptoms	4 (4.8%)	39 (26.0%)	22 (26.2%)	<0.0001	0.0001
Dizziness	22 (26.5%)	14 (9.3%)	8 (9.5%)	0.0005	0.0043
Ataxia	27 (32.5%)	62 (41.3%)	37 (44.0%)	0.1854	0.1258
Nystagmus	21 (25.3%)	13 (8.7%)	6 (7.1%)	0.0005	0.0014
Vision impairment	15 (18.1%)	9 (6.0%)	4 (4.8%)	0.0037	0.0068
Hearing impairment	3 (3.6%)	7 (4.7%)	2 (2.4%)	0.7043	0.6818
Somnolence	11 (13.3%)	20 (13.3%)	10 (11.9%)	0.9862	0.7928
Tremor	7 (8.4%)	22 (14.7%)	12 (14.3%)	0.1675	0.2337
Speech disturbance	15 (18.1%)	37 (24.7%)	24 (28.6%)	0.2470	0.1088
Memory impairment	37 (44.6%)	19 (12.7%)	16 (19.0%)	<0.0001	0.0004
Attention disorder	14 (16.9%)	2 (1.3%)	2 (2.4%)	<0.0001	0.0015
Paresis/plegia	22 (26.5%)	47 (31.3%)	29 (34.5%)	0.4396	0.2607
Hallucinations	9 (10.8%)	2 (1.3%)	2 (2.4%)	0.0019	0.0275
Emotional changes	27 (32.5%)	5 (3.3%)	5 (6.0%)	<0.0001	<0.0001
Rash	1 (1.2%)	14 (9.3%)	8 (9.5%)	0.0155	0.0173
Pelvic organ dysfunction	10 (12.0%)	5 (3.3%)	4 (4.8%)	0.0094	0.0894
Laboratory data					
WBC (×10^9^/L, serum)	7.88 (5.84–10.30)	8.98 (6.50–12.85)	8.38 (6.39–10.22)	0.0451	0.5000
CRP (mg/L, serum)	2.12 (0.60–5.75)	11.40 (2.00–85.00)	4.15 (1.06–21.31)	<0.0001	0.0124
Cell count (cells/μL, CSF)	5 (2–18.75)	121 (43.75–410.75)	61 (29.75–126.75)	<0.0001	<0.0001
Protein (g/L, CSF)	0.46 (0.32–0.71)	1.02 (0.60–2.07)	0.73 (0.49–1.15)	<0.0001	<0.0001
Glucose (mmol/L, CSF)	3.51 (3.31–4.03)	3.00 (2.45–3.71)	3.24 (2.82–3.94)	0.0001	0.0300
Oligoclonal bands (CSF)	15/44 (34.1%)	14/34 (41.2%)	10/26 (38.5%)	0.5208	0.7123
EEG data					
Diffuse slowing/non-epileptic abnormalities	31/65 (47.7%)	49/60 (81.7%)	33/41 (80.5%)	<0.0001	0.0008
Epileptic abnormalities	21/65 (32.3%)	15/60 (25.0%)	12/41 (29.3%)	0.3674	0.7421
MRI abnormalities					
White matter lesions	4 (4.8%)	22 (14.7%)	11 (13.1%)	0.0294	0.0762
Basal ganglia	6 (4.8%)	13 (8.7%)	3 (3.6%)	0.7799	0.3175
Corpus callosum	1 (1.2%)	7 (4.7%)	2 (2.4%)	0.2684	0.5966
Pontine	2 (2.4%)	4 (2.7%)	3 (3.6%)	0.9515	0.7004
Midbrain	2 (2.4%)	4 (2.7%)	3 (3.6%)	0.9515	0.7004
Thalamus	5 (6.0%)	10 (6.7%)	6 (7.1%)	0.9218	0.8360
Corona radiata	1 (1.2%)	8 (5.3%)	3 (3.6%)	0.1690	0.6211
Cortical edema	4 (4.8%)	3 (2.0%)	3 (3.6%)	0.2375	0.7134
Cerebellum	2 (2.4%)	6 (4.0%)	3 (3.6%)	0.7178	0.7004
Limbic system	29 (34.9%)	42 (28.0%)	30 (35.7%)	0.1756	0.8949
Contrast enhancement	10/74 (13.5%)	53/113 (46.9%)	24/71 (33.8%)	<0.0001	0.0039
Edema	9 (10.8%)	37 (24.7%)	25 (29.8%)	0.0172	0.0039
Restriction on DWI	9 (10.8%)	35 (23.3%)	14 (16.7%)	0.0292	0.3337

IQR: interquartile range; GI: gastrointestinal; WBC: white blood cells; CRP: c-reactive protein; CSF: cerebrospinal fluid; EEG: electroencephalography; MRI: magnetic resonance imaging; DWI: diffusion-weighted imaging. ^†^ The infectious group includes both bacterial and viral cases; * *p*-values represent comparisons between the autoimmune and infectious groups; ** *p*-values represent comparisons between the autoimmune and viral groups.

**Table 3:** Features used by humans for diagnostic decisions and features selected for AI modeling after RFECV.

Features	Used by Humans	Selected for AI Model (After RFECV)
Demographics and clinical	X	
Age	X	X
Sex_male	X	X
Rash	X	
Headache	X	X
Fatigue	X	
Sleep impairment	X	
Gait disturbance	X	
Behavioral changes	X	
Shivering	X	
Balance disorder	X	X
Catatonia	X	
Fever	X	X
Consciousness disturbance	X	X
Joint/muscle pain	X	
Dyspnea	X	
Seizures	X	X
Drooling	X	
Cough	X	
Myoclonus	X	
Sore throat	X	
Disorientation	X	
Paresthesia	X	
Fainting	X	
GI symptoms	X	
Back pain	X	
Chills	X	
Dizziness	X	
Ataxia	X	X
Nystagmus	X	X
Visual impairment	X	X
Hearing impairment	X	
Lethargy	X	X
Somnolence	X	
Tremor	X	
Delirium	X	
Dysphagia	X	
Speech disorder	X	
Memory impairment	X	X
Attention disorder	X	X
Paresis	X	X
Hallucinations	X	
Emotional changes	X	X
Olfactory disturbance	X	
Pelvic organ dysfunction	X	X
Laboratory features	X	
WBC_serum	X	X
CRP_serum	X	X
Cell count_CSF	X	X
Protein_CSF	X	X
Glucose_CSF	X	X
Oligoclonal bands_CSF	X	
EEG		
Diffuse slowing/non-epileptic abnormalities	X	X
Focal epileptic abnormalities	X	
MRI features		
Leukoencephalopathy	X	X
Basal ganglia	X	X
Cerebellar peduncles	X	
Corpus callosum	X	
Pontine	X	
Midbrain	X	
Thalamus	X	
Cortical edema	X	
Corona radiata	X	
Cerebellum	X	
Limbic system	X	
Enhancement_MRI	X	
Enhancement_leptomeningeal	X	
Enhancement_pachymeningeal	X	
Enhancement_linear	X	
Restricted diffusion_DWI	X	X
Edema_MRI	X	X

RFECV: recursive feature elimination with cross-validation; EEG: electroencephalography; MRI: magnetic resonance imaging; DWI: diffusion-weighted imaging; WBC: white blood cell; CRP: C-reactive protein; CSF: cerebrospinal fluid; X: feature considered for inclusion.

**Table 4:** Performance metrics of machine learning models for the diagnosis of encephalitis.

Model	Accuracy	Precision	Sensitivity	Specificity	F1-Score	AUROC
Random Forest	0.971	1.000	0.920	1.000	0.958	0.966
XGBoost	0.943	0.957	0.880	0.978	0.917	0.940
LightGBM	0.943	0.957	0.880	0.978	0.917	0.949
Logistic Regression	0.943	0.920	0.920	0.956	0.920	0.964
Naïve Bayes	0.886	0.840	0.840	0.911	0.840	0.880
K-nearest Neighbors	0.871	0.833	0.800	0.911	0.816	0.865

**Table 5:** Performance comparison of the AI model and human evaluators in classifying encephalitis cases.

	Accuracy	Precision	Sensitivity	Specificity	F1-Score	AUROC
AI model	0.971	1.000	0.920	1.000	0.958	0.966
Neurologist in training 1	0.900	0.846	0.880	0.911	0.863	0.896
Neurologist in training 2	0.800	0.677	0.840	0.778	0.750	0.809
Neurologist in training 3	0.757	0.618	0.840	0.711	0.712	0.776
Attending physician 1	0.843	0.733	0.880	0.822	0.800	0.851
Attending physician 2	0.829	0.933	0.560	0.978	0.700	0.769
Attending physician 3	0.871	0.864	0.760	0.933	0.809	0.847

## Data Availability

The datasets generated and/or analyzed in the current study are available from the corresponding author upon reasonable request.

## Supplementary Materials

The following supporting information can be downloaded at https://www.mdpi.com/article/10.3390/jcm14228222/s1, Table S1. Hyperparameter search spaces for the machine learning classifiers, Figure S1. SHAP force plots illustrating the impact of clinical features on model predictions for autoimmune encephalitis cases.
