# Supplementary material for: Differential Diagnosis of Infectious Versus Autoimmune Encephalitis Using Artificial Intelligence-Based Modeling
Source: J Clin Med. 2025 Nov 20;14(22):8222. doi: 10.3390/jcm14228222 (PMC12653740; doi:10.3390/jcm14228222)
Supplement: Supplementary file 1 [file jcm-14-08222-s001.zip › Supplementary material S1.pdf]

Supplementary material S1

Table S1 Hyperparameter search spaces for the machine learning classifiers

Model	Hyperparameters
Random Forest	n_estimators = [100, 200, 300]<br>max_depth = [3, 5, 7, None]<br>max_features = ['auto', 'sqrt', 'log2']<br>min_samples_split = [2, 5, 10]<br>min_samples_leaf = [1, 2, 4]
XGBoost	n_estimators = [100, 200, 300]<br>max_depth = [3, 5, 7]<br>learning_rate = [0.01, 0.1, 0.2]<br>subsample = [0.7, 0.8, 1.0]<br>colsample_bytree = [0.7, 0.8, 1.0]
LightGBM	n_estimators = [100, 200, 300]<br>learning_rate = [0.01, 0.1, 0.2]<br>num_leaves = [31, 50, 70]<br>subsample = [0.7, 0.8, 1.0]<br>colsample_bytree = [0.7, 0.8, 1.0]
Logistic Regression	C = [0.01, 0.1, 1, 10, 100]<br>penalty = ['l1', 'l2']
K-Nearest Neighbors	n_neighbors = [3, 5, 7, 9]<br>weights = ['uniform', 'distance']<br>metric = ['euclidean', 'manhattan']
Gaussian Naive Bayes	No hyperparameters tuned
