# Supplementary material for: Differential Diagnosis of Infectious Versus Autoimmune Encephalitis Using Artificial Intelligence-Based Modeling
Source: J Clin Med. 2025 Nov 20;14(22):8222. doi: 10.3390/jcm14228222 (PMC12653740; doi:10.3390/jcm14228222)
Supplement: Supplementary file 1 [file jcm-14-08222-s001.zip › Supplementary material S2.pdf]

Supplementary material S2

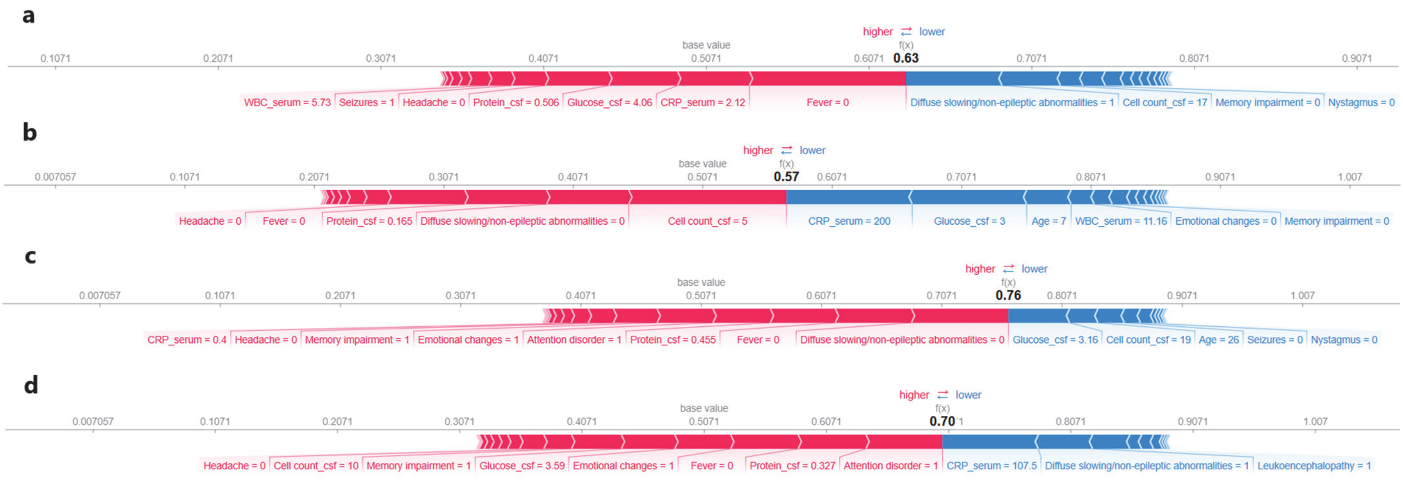

Figure S1 SHAP force plots illustrating the impact of clinical features on model predictions for autoimmune encephalitis cases. (a) LGI1-antibody encephalitis, (b) NMDAR-antibody encephalitis, (c) and (d) seronegative encephalitis cases. Features pushing the prediction higher are shown in red, while features pushing the prediction lower are shown in blue
